# Body Composition in Multiple Sclerosis Patients and Its Relationship to the Disability Level, Disease Duration and Glucocorticoid Therapy

**DOI:** 10.3390/nu14204249

**Published:** 2022-10-12

**Authors:** Edyta Matusik, Jacek Durmala, Barbara Ksciuk, Pawel Matusik

**Affiliations:** 1 Department of Rehabilitation, Faculty of Health Sciences in Katowice, Medical University of Silesia, 40-055 Katowice, Poland; 2 Multiple Sclerosis Management Center “Novomed”, 40-650 Katowice, Poland; 3 Department of Pediatrics, Pediatric Obesity and Metabolic Bone Diseases, Faculty of Medical Sciences in Katowice, Medical University of Silesia, 40-055 Katowice, Poland

**Keywords:** multiple sclerosis, body composition, BMI, waist-to-height ratio, glucocorticoid therapy

## Abstract

Background: Patients with multiple sclerosis (MS) have many potential factors (spasticity, immobilization, glucocorticoids use) for the deterioration of body composition. Aim: To assess the nutritional status (by classical anthropometry and by bioelectrical impedance analysis (BIA)) in MS patients and to correlate it with clinical state, MS duration time and the presence of glucocorticoid therapy in anamnesis (ever used). Methods: Anthropometrical (BMI and waist and hip circumferences, waist-to-height ratio (W/HtR), and waist-to-hip ratio (WHR)) and body composition (BIA) data were evaluated in 176 patients with MS. Fat mass (FM), and fat-free mass (FFM) were expressed as kilograms (kg), percentage (%) and indexes (FMI: fat mass index, FFMI: fat-free mass index) expressed in kg/m^2^. The median Expanded Disability Status Scale score was 4.5. Patients were then divided according to EDSS score as mild (EDSS 1.0–4.0) or moderate (EDSS 4.5–6.5) disability subgroup. Results: Waist c., WHtR, WHR, and FM% were significantly higher in the moderate MS group (*p* < 0.01; *p* < 0.001; *p* < 0.001; and *p* < 0.05, respectively). Whilst, FFM% was significantly lower (*p* < 0.05). BMI did not correlate significantly with any disability status score and MS time. Significant correlations were observed between EDSS, ΔEDSS and MS time and Waist c., WHtR, WHR, FM% and FFM%. WHtR had the strongest significance (*p* < 0.0001 vs. EDSS; *p* < 0.0001 vs. ΔEDSS; and *p* < 0.01 vs. MS time, respectively). After the adjustment to the MS time, only FM% was no longer significantly related to both EDSS and ΔEDSS. MS duration time, EDSS, ΔEDSS, WHtR, FM(kg), FM%, and FMI were significantly higher in the patients with a positive history of glucocorticoid therapy (all *p* < 0.05). Whilst, FFM% was significantly lower in MS patients treated with glucocorticoids (*p* < 0.01). Conclusions: Greater disability in MS patients is strongly related to lower fat-free mass and higher fat mass, especially with the abdominal distribution, irrespective of the duration time of the disease. Oral glucocorticoid therapy seems to have a negative impact on the body composition of MS patients. However, further prospective multifactorial studies in this field have to be done. For the proper assessment of nutritional status in MS patients, Waist c., WHtR, WHR, or body composition parameters seem to be of greater use than BMI.

## 1. Introduction

Multiple sclerosis (MS) is an autoimmune disorder that is characterized by the diffuse demyelination process of the central nervous system (CNS) caused by inflammation [1]. Spasticity, weakness and fatigue, impaired coordination, reduced mobility and ambulation, abnormal sensation (e.g., dysesthesia, paresthesia, and proprioceptive feeling), sexual dysfunction, and depression are the main symptoms of MS. The most popular theory of MS etiology is that the disease is provoked by the complex interaction between the genetic background and the environmental factors which can influence both the pathogenesis and progression of the disease. Some of them seem to be modifiable, which may have practical importance for the treatment and prognosis of MS outcomes. One of the recently underlined modifiable factors is excessive body weight [2,3,4,5]. A large-scale population-based study conducted by Hedström et al. showed that persons whose BMI exceeded 27 kg/m^2^ at age 20 had a two-fold increased risk of developing MS compared with normal-weight subjects in the Swedish population [4]. Moreover, a study conducted by Cambil-Martin et al. [6] showed that overweight MS patients had higher depression levels, lower functional capacity, and worse self-rated health status in comparison to normal-weight MS patients. In the recently published review, the authors performed an assessment of the available data from the last 10 years [5]. They found a causal relationship between childhood and adolescent obesity and MS. In the second part, the pathophysiological mechanisms that may explain the correlations between obesity and MS, focusing mainly on the adipokines, were described. However, the authors underline that no relevant data were found regarding the association between obesity and disability (high EDSS score) in MS patients. Thus, they consider that this topic should be elucidated in future studies. However, our recently published study confirmed the significant correlation between anthropometrical parameters (waist-to-hip ratio, WHtR, fat mass, and fat-free mass) and disability level (EDSS) in MS patients [7]. Moreover, body mass index (BMI), which is the most widely used for nutritional status assessments, was not related to the EDSS. The limitation of BMI’s use in MS patients was described by Pilutti et al. [8], and in the review by Dionyssiotis [9], which reported that BMI assessment might underestimate adiposity in patients with multiple sclerosis. For the proper body composition analysis (fat mass, fat-free mass, and muscle mass), the two most widely methods: dual energy X-ray absorptiometry (DXA) or bioelectrical impedance analysis (BIA)), have to be performed. However, body composition assessment in people with MS has not been extensively studied. Moreover, patients with multiple sclerosis (MS) have many other potential factors (disease duration time, spasticity, immobilization, or glucocorticoid use) which can deteriorate the anthropometrical status and body composition and may have the potential impact on both the progression and prognosis of the disease. That is why the aim of this study was to assess the nutritional status (by classical anthropometry and by bioelectrical impedance analysis (BIA)) in MS patients and to correlate it with disability state, MS duration time and the presence of glucocorticoid therapy in anamnesis.

## 2. Materials and Methods

### 2.1. Studied Population

195 MS patients (132 females/63 males) that were consecutively admitted to the Multiple Sclerosis Management Center were recruited to the study. Subjects who had not experienced an exacerbation within the 30 past days and had no medical conditions such as cardiological diseases, endocrine disorders, diseases of the musculoskeletal system, current glucocorticoid therapy, or respiratory diseases were included. The patients who met the criteria consisted final study group (*n* = 176, 128 females/48 males, age 45.68 ± 12.01 years). All the patients had a definite diagnosis of relapsing-remitting (RRMS) or secondary progressive (SPMS) multiple sclerosis according to the McDonald criteria [10] based on the Expanded Disability Status Scale (EDSS) and preservation of at least some ambulatory function (EDSS < 7.0). The EDSS range was 1.0–6.5, with a median score of 4.5. Initial EDSS was taken retrospectively from the medical history at the moment of the MS diagnosis. The assessment of neurological status on the EDSS was performed by 2 experienced and Neurostatus-certified neurologists (EM, BK). Patients were then divided according to EDSS score as mild (EDSS 1.0–4.0) or moderate (EDSS 4.5–6.5) disability subgroup.

### 2.2. Anthropometric Measurements and Body Composition Analysis

A set of anthropometric measurements was recorded on the day of the visit and was made by a well-trained anthropometrist (PM). Standing height was measured by a wall-mounted Harpenden Stadiometer (Model 602VR, Holtain, Wales, UK) to the nearest 0.1 cm. Weight (in underwear) was measured with an electronic scale with readings accurate to 0.1 kg. Body mass index (BMI) was then calculated using the standard formula (kilograms per meter squared (kg/m^2^)). Waist and hip circumferences were also measured, and waist-to-hip ratio (WHR) and waist-to-height ratio (W/HtR) were then calculated. Body composition parameters: fat mass (FM) and fat-free mass (FFM) were assessed (in kilograms [kg] or as a percentage of body weight [%]) were based on bioelectrical impedance using a leg-to-leg body composition analyzer (BC-420 MA Tanita Europe BV, Hoofddorp, The Netherlands) [11]. Fat mass and fat-free mass indexes were also calculated (FMI and FFMI, respectively) and expressed in kg/m^2^. All the anthropometrical and body composition parameters were measured at the same time point as the subsequent EDSS assessment. The summary of clinical and anthropometrical characteristics of the study group is presented in Table 1. Clinical status both at the diagnosis and subsequent (expressed as EDSS) and disease progression (expressed as ΔEDSS) did not differ significantly between females and males. However, typical significant differences related to gender in the majority of anthropometrical and body composition parameters were noted.

### 2.3. Ethical Considerations

The study was approved by the Ethics Committee of the Medical University of Silesia (Approval No. KNW/0022/KB/179/17). All participants gave informed consent. Patient rights were also approved according to the Helsinki Declaration.

### 2.4. Statistical Analysis

All data were distributed normally (assessed by Kolmogorov–Smirnov test). Differences in continuous variables were assessed by paired Student’s *t*-test for independent variables with non-equal variances. Correlations between continuous parametrical were based on linear Pearson’s correlation coefficient. To be able to find the independent relations between anthropometrical measures, body composition variables, MS duration time and disability status (EDSS), the multiple linear regression analysis was performed when EDSS was a linear dependent variable. All statistical analysis was made with the Statistica™ 12 PL software, and a *p*-value less than 0.05 was considered statistically significant.

## 3. Results

### 3.1. Anthropometrical Measures and Body Composition Variables by Disability Status

Clinical characteristics, anthropometrical measures and body composition variables for the overall group and by disability status are presented in Table 2. Age, MS duration time, initial EDSS, EDSS and ΔEDSS were significantly higher in the moderate than mild disability group, as expected (all *p* < 0.0000001). From classical anthropometry Waist c., WHtR and WHR were significantly higher in the moderate than mild disability group (95.9 vs. 88.7 *p* < 0.01; 0.58 vs. 0.53 *p* < 0.001 and 0.94 vs. 0.88 *p* < 0.001 respectively). However, there were no significant differences in body weight, body mass and BMI between the disability group. From the body composition variables, only FM% and FFM% differ significantly in opposite directions in the moderate vs. mild disability group (all *p* < 0.05). Meanwhile, FM and FFM expressed in kg and as indexes did not differ significantly between the disability groups.

Similar significant differences between both clinical and anthropometrical measures were found after stratification for the MS phenotype (Table 3). Patients with SPMS had significantly higher levels of adiposity, higher visceral fat deposition and higher body mass assessed by BMI.

Fat tissue distribution and body composition parameters are related to gender. That is why the same analysis was made for both the female and male subgroups. Similar differences between mild and moderate MS subgroups were noted either for females or males; however, for males, the majority of differences were not statistically significant (Table 4).

### 3.2. Correlation between Disability Status, MS Duration Time and Anthropometrical Measures/Body Composition Variables

Pearson’s correlations between disability scores (Initial EDSS, EDSS and ΔEDSS), MS time and nutritional status (anthropometrical measures/body composition variables) in the overall study group are presented in Table 5. There were no significant correlations between initial EDSS and any evaluated nutritional status describing parameters. BMI did not correlate significantly with any disability status score and MS time. Significant correlations were observed between EDSS, ΔEDSS and MS time and anthropometrical measures describing abdominal fat distribution (Waist c., WHtR, WHR). However correlation with WHtR had the strongest significance (r = 0.293 *p* < 0.0001 vs. EDSS; r = 0.308 *p* < 0.0001 vs. r = 0.229 ΔEDSS; and *p* < 0.01 vs. MS time, respectively). From body composition variables, only FM% and FFM% correlated significantly with present EDSS, ΔEDSS and MS time (FM% in a positive and FFM% in a negative manner).

Since anthropometrical measures/body composition variables correlated with MS time in the same manner as between disability status scores (EDSS and ΔEDSS), a multiple linear regression analysis was performed. After the adjustment to the MS time, both EDSS and ΔEDSS were still significantly associated with abdominal fat tissue parameters (Waist c., WHtR and WHR). However, there was only a significant negative correlation between disability scores and FFM%, whilst the association between both EDSS and ΔEDSS vs. FM% was no longer significant (Table 6).

### 3.3. Anthropometrical Measures, Body Composition Variables and MS Duration Time by Glucocorticoid Therapy

Clinical characteristics, anthropometrical measures and body composition variables for the overall group and by glucocorticoid therapy are presented in Table 7. Glucocorticoid therapy duration was taken retrospectively from the available medical documentation. MS duration time, EDSS and ΔEDSS were significantly higher in the patients who were treated by oral glucocorticoid in anamnesis (all *p* < 0.05). From classical anthropometry, only WHtR was significantly higher in the treated vs. non-treated subgroup (*p* < 0.05). However, there were no significant differences in BMI, Waist c., Hip c., and WHR between subgroups stratified for glucocorticoid therapy. From the body composition variables, every fat mass describing parameters (FM(kg); FM% and FMI) were significantly higher in the group treated by glucocorticoids (all *p* < 0.05). From the variables related to the fat-free mass, only FFM% was significantly lower in MS patients with a positive history of glucocorticoid therapy (68.86 vs. 72.31 *p* < 0.01).

## 4. Discussion

In our study, we conducted an assessment of standard anthropometrical measurements and body composition in patients with MS with respect to disability status, duration of the disease and glucocorticoid therapy in the medical history. We observed that, from classical anthropometry, only the measures related to abdominal fat tissue distribution (Waist c., WHtR, and WHR) were significantly higher in persons with MS with moderate disability. However, BMI, the most widely used anthropometrical parameter for the assessment of nutritional status, did not differ significantly between disability status groups. From the body composition variables, fat mass (FM) and fat-free mass (FFM) expressed as a percentage of body mass differ significantly in opposite directions between disability groups. Our findings confirm the data from the study by Pilutti et al. [12], which showed that FM assessed by DXA was significantly higher in MS patients with moderate disabilities. Moreover, they also did not find a significant difference in BMI between the mild vs. moderate disability groups. In another paper by the same authors, using data coming from body composition measured by DXA, BMI assessments may cause an underestimation of adiposity in patients with MS [4]. The same conclusions were drawn by Wingo et al. [13], which showed a significantly higher fat mass and lower fat-free mass in men with MS compared to healthy BMI-matched controls. The limitation of BMI use in MS patients was also presented in the study showing the relationship between MS and longitudinal changes in BMI [14]. The authors made three main observations. Baseline BMI in MS patients was significantly higher compared to healthy control, BMI was significantly higher in healthy controls with increasing age, and there were no longitudinal associations between BMI and EDSS. A recently published study postulated the use of a simple model to estimate the percentage of body fat in persons with MS, based only on BMI and sex, using a special mathematic formula. However, it was cross-validated with DXA body composition only in 33 MS patients (six males) and not related to the disability status [15].

Dual-energy X-ray absorptiometry (DXA) is currently the gold standard, not only for the diagnosis of osteoporosis but also for fat mass evaluation. However, a noninvasive body composition assessment technique is currently available based on bioelectrical impedance analysis (BIA). A good correlation between BIA and DXA has been reported in estimating both fat mass and fat-free mass in different populations [16,17]. BIA is a relatively inexpensive, quick, simple, readily accessible and noninvasive technique. Body composition analysis by BIA in MS patients has been used in the two studies, which focused mainly on nutritional intake rather than nutritional status [18,19]. The MS patient groups were relatively small (*n* = 20 and *n* = 37 respectively), and MS patients and body fat percentage assessed by BIA were only shown as a mean result for the studied population, but the authors did not show any detailed results related to body composition.

In the present study, we found a significant correlation between disability status (EDSS, ΔEDSS) and MS time vs. anthropometrical measures describing abdominal fat distribution (Waist c., WHtR, WHR). There were no significant correlations between initial EDSS and any evaluated nutritional status describing parameters. BMI did not correlate significantly with any disability status score and MS time which confirmed results coming from the different studies about the limitation of this parameter use in MS patients [12,14]. WHtR had the strongest relationship with disability status from all simple anthropometry measures. There were also significant correlations between body composition variables (FM% and FFM%) with both disability status and MS time in an opposite manner. These findings confirmed our recently published data with the significant correlation between anthropometrical/body composition parameters (WHR, WHtR, FM% and FFM%) and disability status (EDSS) in MS patients [7]. However, conflicting results of lack of significant correlations between EDSS vs. BMI, Waist c., WHR, and FM% from BIA were noted in a group of 137 Brazilian MS patients [20]. To be able to adjust our results from the MS duration time, multiple linear regression analysis was made. After the adjustment to the MS time, both EDSS and ΔEDSS were still significantly related to abdominal fat tissue parameters (Waist c., WHtR and WHR). However, there was only a significant negative correlation between disability scores and FFM%, whilst correlations between both EDSS and ΔEDSS vs. FM% were no longer significant. This underlines the importance of lean/muscle mass in the prognosis of the development of the diseases.

Body composition parameters assessment seems to be important because, in MS patients, the possible higher risk for sarcopenia related to the level of disability is very high. A study conducted by Wens et al. [21] revealed a higher fat percentage and a lower lean mass from muscle biopsies in MS patients. Other data published by Ward et al. [22] and Wingo et al. [13] showed that a higher FM% and lower FFM% were associated with lower-limb physical function, suggesting that body composition, specifically reducing adiposity and increasing lean mass may be a potential target for MS interventions. It is also important to realize that lean mass is strongly related to bone mineral density (BMD), either in the whole body or lumbar spine projections [23]. Our findings also confirm the great importance of body fat distribution, with the special risk for abdominal/visceral obesity in MS patients, which cannot be properly assessed using BMI itself. WHtR is now widely studied, with the aim of finding relatively simple parameters of fat tissue distribution in connection with visceral obesity and its comorbidities. A recent analysis showed that WHtR seems to be the best parameter for the prognosis of visceral fat and its comorbidities [24]. The other aspect of the potential adiposity influence on the progression of MS is the generation of adipose tissue-related inflammation and oxidative stress. A recent study by Drehmer et al. [25] revealed a significant correlation between fat mass distribution assessed by both waist circumference and WHtR with oxidative stress and inflammation markers in obese patients with MS.

The other authors showed a significant relationship between the different adiposity indexes (Body Shape Index (ABSI), the Body Roundness Index (BRI), and the Visceral Adiposity Index (VAI)) with plasma lipid profiles in patients with MS [26]. That’s why the relationship between body composition and possible metabolic syndrome markers has to be a matter for future research.

Based on our study, MS duration time, EDSS and ΔEDSS were, as expected, significantly higher in the patients who had a positive medical history of glucocorticoid therapy. From classical anthropometry, only WHtR was significantly higher in the treated vs. non-treated subgroup. This result confirms one more time the practical usefulness of this parameter in daily practice. From the body composition variables, every fat mass describing parameters (FM(kg); FM% and FMI) and FFM% were significantly higher in the group treated by glucocorticoids. In the study conducted by Formica et al. [27], a significant reduction of fat-free mass content in women with MS compared to healthy controls was noted in body composition assessment by DXA. The authors noted that the main factor related to this difference was glucocorticoid therapy. Glucocorticoid therapy is also strongly related to reduced bone mineral density in MS patients, even in relatively low cumulative doses [28]. Our data confirm that glucocorticoid therapy is a negative factor related to adiposity in MS patient and have to be taken into account in the diagnostic and therapeutic procedures in the MS population. However, it is well known that glucocorticoid therapy has a direct effect on the hydration of the patients by inducing both water and sodium retention by promoting sodium reabsorption at the level of the renal tubule. That’s why it can affect the estimation of FFM by BIA because the increased hydration of soft tissue could lead to an overestimation of FFM and, consequently, an underestimation of FM.

The limitations of this study must be acknowledged. First was the lack of data describing the initial nutritional status of analyzed patients. Second, we have to take into account that patients with better clinical status (lower EDSS scores) may have more social (dietary and behavioral) opportunities to change their body composition by interaction with friends and family and at work, which allows for more frequent episodes of dining outside of the house and quite normal daily physical activity. In contrast, participants with higher EDSS scores may be dependent on a caretaker for meal preparation and definitely have lower physical activity, and probably have a higher risk for body composition deterioration (adiposity or sarcopenia). Other limits are the EDSS’s dependence on age and disease duration. However, the linear regression analysis showed that anthropometrical parameters and body composition variables are significantly associated with clinical state irrespectively from the duration of the diseases. All these limitations indicate that future studies will have to focus on the prospective longitudinal body composition assessment in MS patients. Moreover, specific MS severity subgroups of the patients and other potentially confounding factors have to be taken into account.

## 5. Conclusions

Greater disabilities in MS patients are significantly associated with lower fat-free mass and higher fat mass, especially with the abdominal distribution, irrespective of the duration time of the disease. Oral glucocorticoid therapy seems to be one of the negative risk factors influencing the body composition of MS patients. However, due to the cross-sectional character of the study and a lot of possible confounding factors (lack of initial anthropometrical data), further multifactorial prospective analyses are needed. For proper assessment of nutritional status in patients with MS, Waist c., WHtR, WHR or body composition analysis instead of BMI should be used.

## Figures and Tables

**Table 1:** Clinical and anthropometrical characteristics of the study population.

	Studied Population *n* = 176 (F/M 128/48)			
	Mean	Minimum	Maximum	SD
Age (years) [F/M]	45.68 [45.67/45.71]	20.00	73.00	12.01
MS duration (years) [F/M]	10.92 [10.60/11.80]	0.17	37.00	7.91
EDSS initial [F/M]	2.2 [2.2/2.2]	1.0	4.5	0.7
EDSS [F/M]	3.3 [3.3/3.3]	1.0	6.5	1.6
Height (cm) [F/M]	167.2 [163.8/176.3] **	152.1	196.1	8.7
Weight (kg) [F/M]	69.5 [65.3/80.8] **	40.6	114.	16.0
BMI (kg/m^2^) [F/M]	24.87 [24.4/25.9]	16.10	40.30	4.94
Waist c. (cm) [F/M]	90.4 [87.9/97.1] **	61.0	126.0	13.5
Hip c. (cm) [F/M]	100.9 [100.6/101.9]	73.5	129.0	9.3
WHtR [F/M]	0.54 [0.54/0.55]	0.38	0.75	0.08
WHR [F/M]	0.89 [0.87/0.96] **	0.66	1.22	0.08
FM (kg) [F/M]	20.7 [21.1/19.6]	2.6	52.7	9.2
FMI (kg/m^2^) [F/M]	7.43 [7.83/6.36] *	1.02	18.90	3.33
FM (%) [F/M]	28.8 [30.8/23.6] **	6.2	48.1	8.4
FFM (kg) [F/M]	48.9 [44.3/61.1] **	34.8	75.6	10.1
FFMI (kg/m^2^) [F/M]	17.35 [16.1/19.6] **	13.21	23.28	2.33
FFM (%) [F/M]	71.2 [69.2/76.3] **	51.9	93.8	8.3

Abbreviations: EDSS—Expanded Disability Status Scale; BMI—body mass index; WHtR—waist-to-height ratio; WHR—waist-to-hip ratio; FM—fat mass; FMI—fat mass index; FFM—fat-free mass; FFMI—fat-free mass index; * *p* < 0.001; ** *p* < 0.0001 (females vs. males).

**Table 2:** Clinical and anthropometrical characteristics of the overall study population with MS and by the level of disability. Results are expressed as means (SDs).

	Overall (*n* = 176)	Mild (*n* = 135)	Moderate (*n* = 41)	*p*-Value Student’s *t*-Test
Age (years)	45.68 (12.01)	42.88 (11.19)	54.90 (9.09)	<0.0000001
MS duration (years)	10.92 (7.91)	9.29	16.30	<0.0000001
Initial EDSS	2.2 (0.7)	2.0 (0.7)	2.7 (0.6)	<0.000001
Present EDSS	3.3 (1.6)	2.5 (0.9)	5.8 (0.6)	<0.0000001
ΔEDSS	1.1 (1.5)	0.5 (0.9)	3.2 (0.9)	<0.0000001
Height (cm)	167.2 (8.7)	167.3 (8.5)	165.8 (9.0)	NS
Weight (kg)	69.5 (16.0)	69.2 (16.9)	70.3 (12.9)	NS
BMI (kg/m^2^)	24.87 (4.94)	24.60 (5.00)	25.73 (4.70)	NS
Waist c. (cm)	90.4 (13.5)	88.7 (13.4)	95.9 (12.4)	<0.01
Hip c. (cm)	100.9 (9.3)	100.5 (8.6)	102.5 (9.4)	NS
WHtR	0.54 (0.08)	0.53 (0.07)	0.58 (0.08)	<0.001
WHR	0.89 (0.08)	0.88 (0.08)	0.94 (0.10)	<0.001
FM (kg)	20.7 (9.2)	7.2 (3.3)	8.3 (3.2)	NS
FM (%)	28.8 (8.4)	28.1 (8.5)	31.4 (7.4)	<0.05
FMI (kg/m^2^)	7.43 (3.33)	7.16 (3.34)	8.29 (3.20)	NS
FFM (kg)	48.9 (10.1)	49.10 (10.37)	48.07 (8.96)	NS
FFM (%)	71.2 (8.3)	71.9 (8.4)	68.6 (7.6)	<0.05
FFMI (kg/m^2^)	17.35 (2.33)	17.33 (2.34)	17.41 (2.29)	NS

Abbreviations: EDSS—Expanded Disability Status Scale; BMI—body mass index; WHtR—waist-to-height ratio; WHR—waist-to-hip ratio; FM—fat mass; FMI—fat mass index; FFM—fat-free mass; FFMI—fat-free mass index. NS—non-significant.

**Table 3:** Clinical and anthropometrical characteristics of the overall study population with MS and stratified by MS phenotype. Results are expressed as means (SDs).

	Overall (*n* = 176)	RRMS (*n* = 123)	SPMS (*n* = 53)	*p*-Value Student’s *t*-Test
Age (years)	45.68 (12.01)	40.87 (9.69)	56.85 (9.13)	<0.0000001
MS duration (years)	10.92 (7.91)	8.31 (5.69)	16.99 (8.99)	<0.0000001
Present EDSS	3.3 (1.6)	2.6 (1.2)	5.0 (1.4)	<0.0000001
BMI (kg/m^2^)	24.87 (4.94)	24.36 (5.05)	26.04 (4.52)	<0.05
Waist c. (cm)	90.4 (13.5)	87.9 (13.6)	96.1 (11.5)	<0.001
Hip c. (cm)	100.9 (9.3)	100.3 (9.6)	102.7 (8.5)	NS
WHtR	0.54 (0.08)	0.52 (0.07)	0.58 (0.07)	<0.00001
WHR	0.89 (0.08)	0.87 (0.07)	0.93 (0.08)	<0.00001
FM (kg)	20.7 (9.2)	19.21 (9.4)	22.9 (8.3)	<0.05
FM (%)	28.8 (8.4)	27.6 (8.2)	31.7 (8.0)	<0.01
FMI (kg/m^2^)	7.43 (3.33)	6.96 (3.23)	8.50 (3.34)	<0.01
FFM (kg)	48.9 (10.1)	49.14 (10.62)	48.19 (8.59)	NS
FFM (%)	71.2 (8.3)	72.4 (8.1)	68.2 (8.2)	<0.01
FFMI (kg/m^2^)	17.35 (2.33)	17.27 (2.46)	17.51 (1.97)	NS

Abbreviations: RRMS—relapsing-remitting multiple sclerosis, SPMS—secondary-progressive multiple sclerosis; EDSS—Expanded Disability Status Scale; BMI—body mass index; WHtR—waist-to-height ratio; WHR—waist-to-hip ratio; FM—fat mass; FMI—fat mass index; FFM—fat-free mass; FFMI—fat-free mass index.

**Table 4:** Anthropometrical characteristics of the study population by level of disability adjusted to gender. Results are expressed as means (SDs).

**Females (*n* = 128)**	**Mild (*n* = 99)**	**Moderate (*n* = 29)**	***p*-Value**
BMI (kg/m^2^)	24.13 (5.23)	25.49 (4.96)	NS
Waist c. (cm)	86.2 (13.3)	93.8 (12.8)	<0.01
Hip c. (cm)	99.9 (10.3)	103.0 (8.7)	NS
WHtR	0.52 (0.08)	0.58 (0.08)	<0.01
WHR	0.88 (0.07)	0.91 (0.09)	<0.01
FM (kg)	20.5 (10.2)	23.1 (7.7)	NS
FM (%)	29.9 (8.6)	33.8 (5.5)	<0.05
FMI (kg/m^2^)	7.53 (3.57)	8.84 (3.12)	NS
FFM (kg)	44.5 (6.2)	43.7 (4.6)	NS
FFM (%)	70.1 (8.5)	66.0 (5.6)	<0.05
FFMI (kg/m^2^)	16.46 (1.80)	16.65 (2.10)	NS
**Males (*n* = 48)**	**Mild (*n* = 36)**	**Moderate (*n* = 12)**	***p*-Value**
BMI (kg/m^2^)	25.88 (4.11)	26.33 (4.15)	NS
Waist c. (cm)	95.8 (11.1)	100.9 (10.3)	NS
Hip c. (cm)	102.1 (7.1)	101.3 (7.9)	NS
WHtR	0.54 (0.06)	0.58 (0.08)	NS
WHR	0.94 (0.06)	1.00 (0.09)	<0.05
FM (kg)	19.28 (7.56)	20.70 (7.92)	NS
FM (%)	23.0 (5.9)	25.4 (8.2)	NS
FMI (kg/m^2^)	6.15 (2.37)	6.98 (3.12)	NS
FFM (kg)	61.86 (8.74)	58.72 (7.93)	NS
FFM (%)	71.9 (8.4)	68.6 (7.6)	NS
FFMI (kg/m^2^)	19.71 (2.00)	19.26 (1.61)	NS

Abbreviations: EDSS—Expanded Disability Status Scale; BMI—body mass index; WHtR—waist-to-height ratio; WHR—waist-to-hip ratio; FM—fat mass; FMI—fat mass index; FFM—fat-free mass; FFMI—fat-free mass index.

**Table 5:** Correlations of clinical status and MS duration time with individual anthropometric and body composition parameters in studied population (* *p* < 0.05; ** *p* < 0.01; *** *p* < 0.001; **** *p* < 0.0001).

	BMI	Waist c.	WHtR	WHR	FM (%)	FFM (%)
Initial EDSS	−0.018	0.018	0.038	0.097	0.009	−0.018
EDSS	0.099	0.234 **	0.293 ****	0.287 ***	0.191 *	−0.206 **
ΔEDSS	0.115	0.250 **	0.308 ****	0.275 ***	0.201 **	−0.213 **
MS time	0.146	0.180 *	0.229 **	0.254 **	0.183 *	−0.188 *

Abbreviations: EDSS—Expanded Disability Status Scale; BMI—body mass index; WHtR—waist-to-height ratio; WHR—waist-to-hip ratio; FM—fat mass; FFM—fat-free mass.

**Table 6:** Multiple linear regression analysis (y = EDSS and ΔEDSS).

Y	x					
	Variable	b Coefficient	Significance	Variable	b Coefficient	Significance
EDSS	Waist c.	0.155	*p* < 0.01	MS time	0.442	*p* < 0.0000001
	WHtR	0.195	*p* < 0.01		0.426	
	WHR	0.180	*p* < 0.01		0.422	
	FM%	0.109	*p* = 0.109		0.450	
	FFM%	−0.122	*p* = 0.074		0.447	
ΔEDSS	Waist c.	0.176	*p* < 0.01	MS time	0.432	*p* < 0.0000001
	WHtR	0.215	*p* < 0.01		0.415	
	WHR	0.168	*p* < 0.05		0.419	
	FM%	0.127	*p* = 0.062		0.440	
	FFM%	−0.137	*p* < 0.05		0.438	

Abbreviations: EDSS—Expanded Disability Status Scale; WHtR—waist-to-height ratio; WHR—waist-to-hip ratio; FM—fat mass; FFM—fat-free mass.

**Table 7:** Clinical and anthropometrical characteristics of the study population stratified by glucocorticoid therapy (Student’s *t*-test).

	Glucocorticoid Therapy		
	No (*n* = 117)	Yes (*n* = 59)	*p*-Value (*t*-Student Test)
MS time (years)	9.92 (7.07)	12.91 (9.09)	<0.05
Initial EDSS	2.16 (0.74)	2.19 (0.74)	NS
EDSS	3.07 (1.63)	3.66 (1.62)	<0.05
ΔEDSS	0.91 (1.45)	1.47 (1.41)	<0.05
BMI (kg/m^2^)	24.36 (4.67)	25.87 (5.36)	NS
Waist c. (cm)	89.1 (13.19)	93.0 (13.76)	NS
Hip c. (cm)	100.0 (9.1)	102.8 (9.6)	NS
WHtR	0.53 (0.08)	0.56 (0.08)	<0.05
WHR	0.89 (0.09)	0.90 (0.08)	NS
FM (kg)	19.52 (8.43)	22.98 (10.24)	<0.05
FM (%)	27.71 (8.04)	31.09 (8.59)	<0.05
FMI (kg/m^2^)	6.98 (3.04)	8.31 (3.73)	<0.05
FFM (kg)	49.06 (10.43)	48.46 (9.31)	NS
FFM (%)	72.31 (7.96)	68.86 (8.65)	<0.01
FFMI (kg/m^2^)	17.36 (2.43)	17.32 (2.12)	NS

Abbreviations: EDSS—Expanded Disability Status Scale; BMI—body mass index; WHtR—waist-to-height ratio; WHR—waist-to-hip ratio; FM—fat mass; FMI—fat mass index; FFM—fat-free mass; FFMI—fat-free mass index.
